# LINC00240/miR-155 axis regulates function of trophoblasts and M2 macrophage polarization via modulating oxidative stress-induced pyroptosis in preeclampsia

**DOI:** 10.1186/s10020-022-00531-3

**Published:** 2022-09-24

**Authors:** Hai-Ying Wu, Kan liu, Jing-Li Zhang

**Affiliations:** Department of Obstetrics, Henan Provincial People’s Hospital, Zhengzhou University People’s Hospital, Henan University People’s Hospital, Weiwu Road 7, Zhengzhou, 450003 Henan Province People’s Republic of China

**Keywords:** Preeclampsia, LINC00240, Oxidative stress, Cell pyroptosis, Macrophage polarization, Trophoblasts

## Abstract

**Background:**

This study aimed to investigate the effects of LINC00240/miR-155/Nrf2 axis on trophoblast function and macrophage polarization in the pathogenesis of preeclampsia.

**Methods:**

Bindings between LINC00240, miR-155 and Nrf2 were validated by dual luciferase reporter assay or RNA-immunoprecipitation. Cell proliferation, migration, invasion, and pyroptosis were detected by CCK-8, clone formation, wound healing, Transwell system, and flow cytometry, respectively. Macrophage polarization was tested by flow cytometry. The expression levels of LINC00240, miR-155, Nrf2, and oxidative stress and pyroptosis-related markers in in vitro and in vivo preeclampsia models were analyzed by qPCR, western blot, or ELISA assays. Blood pressure, urine protein levels, liver and kidney damages, and trophoblast markers in placenta tissues were further studied in vivo.

**Results:**

Placenta tissues from preeclampsia patients and animals showed decreased LINC00240 and Nrf2 and increased miR-155 expression levels, and the decreased M2 macrophage polarization. LINC00240 directly bound and inhibited expression of miR-155, which then inhibited oxidative stress-induced pyroptosis, promoting proliferation, migration and invasion abilities of trophoblasts, and M2 macrophage polarization. Inhibition of miR-155 led to increased Nrf2 expression and similar changes as LINC00240 overexpression in trophoblast function and macrophage polarization. Overexpression of LINC00240 in in vivo preeclampsia model decreased blood pressure, urine protein, liver and kidney damages, increased fetal weight and length, and induced trophoblast function and M2 macrophage polarization.

**Conclusion:**

LINC00240 inhibited symptoms of preeclampsia through regulation on miR-155/Nrf2 axis, which suppressed oxidative stress-induced pyroptosis to improve trophoblast function and M2 macrophage polarization. LINC00240 could be a potential therapeutic target for preeclampsia.

**Supplementary Information:**

The online version contains supplementary material available at 10.1186/s10020-022-00531-3.

## Introduction

Preeclampsia is a severe complication during pregnancy which affects around 2–8% of pregnant women globally (Duley 2009). Majority of the preeclampsia patients have hypertension and proteinuria, while in other patients, proteinuria is absent (Gestational Hypertension and Preeclampsia 2020). Preeclampsia leads to many manifestations in both mother and fetus, and may cause serious symptoms such as stroke, seizure, and even death (Armaly et al. 2018). Although pathogenesis of preeclampsia remains largely unclear, findings in the past decade revealed key roles of placenta defect in the development of this disease (Hod et al. 2015). Possibly through hypoxia, oxidative stress and inflammation, placenta defect may lead to damage of placenta which then releases excessive amount of anti-angiogenic factors into maternal circulation, causing clinical manifestations of the preeclampsia (Hod et al. 2015). The pathogenesis of preeclampsia is also associated with dysfunction of the immune system, such as elevated inflammatory cytokines and altered monocyte-macrophage system (Vishnyakova et al. 2019). More pro-inflammatory (M1) and less anti-inflammatory (M2) macrophages were observed in placenta in preeclampsia models, and this abnormal polarization state of macrophages could lead to dysfunction of trophoblasts, which may contribute to the impaired trophoblast invasion and spiral artery remodeling in preeclampsia (Przybyl et al. 2016; Li et al. 2016a; Buckley et al. 2016; Kolben et al. 2018). In addition, induction of M2 macrophage polarization was shown to assist successful pregnancy in a preeclampsia rat model (Li et al. 2018a). It is therefore of great clinical importance to investigate the roles of trophoblast function and macrophage polarization in the treatment of preeclampsia.

As a redox-sensitive transcriptional factor which promotes antioxidant defense in placenta, nuclear factor erythroid 2-related factor-2 (Nrf2) is considered to have protective roles against preeclampsia (Yu et al. 2019; Li et al. 2016b). In addition, Nrf2 was shown to inhibit pyroptosis in vascular endothelial cells (Hu et al. 2018) and rat cortical neurons (Li et al. 2017). Only a few studies have focused on the possible roles of pyroptosis in preeclampsia (Shirasuna et al. 2020; Cheng et al. 2019), and further investigations are required. Previous studies showed that microRNA-155 (miR-155) could bind to and inhibit Nrf2 signaling pathway in lung cancer (Gu et al. 2017) and liver injury (Yang et al. 2018). However, no study has reported roles of miR-155/Nrf2 axis in preeclampsia. Considering the roles of miR-155 (Li et al. 2014) and Nrf2 (Li et al. 2016b) in preeclampsia, we hypothesize that miR-155 might also regulate Nrf2 in preeclampsia and then influence the function of trophoblasts through pyroptosis. This hypothesis could also explain the abnormal polarization of macrophages in placentas since pyroptosis could enhance the secretion of high mobility group box 1 (HMGB1) (Hou et al. 2018), which was found to promote M1 macrophage polarization (Son et al. 2016). It is quite important to clarify the relationship between pyroptosis and macrophage polarization in preeclampsia.

MiR-155 was reported to be significantly up-regulated in placentas from preeclampsia patients and is considered as a potential diagnostic marker of this disease (Azizi et al. 2017). Screening of miRNAs in the plasma of preeclampsia patients also revealed up-regulation of miR-155 levels (Jairajpuri et al. 2017). Results of a case–control study also suggested that serum miR-155 could be used as a sensitive and specific biomarker for the diagnosis of preeclampsia (Gan et al. 2017). In addition, miR-155 could regulate endothelial nitric oxide synthase (eNOS), cyclin D1, and CYR61, and affect the function of trophoblasts (Li et al. 2014; Zhang et al. 2010; Dai et al. 2012; Kim et al. 2017).

Long intergenic non-protein coding RNA 240 (LINC00240) is a potential biomarker for ischemic stroke (Zheng et al. 2019) and has been involved in lncRNA-mRNA crosstalk networks which play key roles in the tumorigenesis of esophageal squamous cell carcinoma (ESCC) (Yang et al. 2016). In addition, LINC00240 was found to promote the progression of cervical cancer through modulation of miR-124-3p/STAT3 pathway (Zhang et al. 2020a). However, the possible roles of LINC00240 in preeclampsia or macrophage polarization have not been reported.

In our study, we looked into the roles of LINC00240, miR-155, and Nrf2 in the pathogenesis of preeclampsia and suspected that LINC00240 could regulate the expression of miR-155, which then modulated Nrf2 expression and inhibited oxidative stress-induced pyroptosis. The inhibited pyroptosis on one hand promoted proliferation, migration and invasion of trophoblasts, and on the other hand promoted M2 and inhibited M1 polarization of macrophages in preeclampsia through decreasing the secretion of HMGB1. Our animal study results also suggested that LINC00240 could be a potential target for the treatment of preeclampsia. On the basis of previous findings, our studies further shed light on the underlying molecular mechanism of preeclampsia, and to the best of our knowledge, our study is the first to show the roles of LINC00240 in preeclampsia.

## Materials and methods

### Clinical sample collection

Human placenta samples were collected from 24 preeclampsia patients and 24 normal pregnant women registered at the Department of Obstetrics, Henan Provincial People’s Hospital (Zhengzhou, Henan, China) after their delivery, and then quickly put into − 80 °C freezer. The clinical characteristics of normal pregnant women and preeclampsia patients were shown in Table 1. The diagnostic criteria of preeclampsia was described as follows: hypertension (systolic blood pressure not lower than 140 mmHg and/or diastolic blood pressure not lower than 90 mmHg) and newly-onset proteinuria (higher than 300 mg per day) (Li et al. 2020; Wang et al. 2019). Exclusion criteria included pre-delivery infection, multiple pregnancies, alcoholism, smoking and alcohol abuse, genetic abnormalities, fetus growth retardation, rupture of membrane, cholestasis of pregnancy, and history of cardiovascular diseases, renal diseases, autoimmune diseases, diabetes, chronic hypertension, or other metabolic diseases. Written informed consent was collected from all participants before the procedure. The research protocol was approved by the Ethical Committee of the Henan Provincial People’s Hospital (Zhengzhou, Henan, China).

**Table 1:** Clinical characteristics of normal and preeclamptic pregnancies

Characteristics	Control (n = 24)	PE (n = 24)
Fetal sex (male)	13	10
Fetal sex (female)	11	14
Primipara (cases)	15	11
Pluripara (cases)	9	13
Maternal age (year)	30.47	31.65
Maternal weight (kg)	66.49	69.89
Systolic blood pressure (mmHg)	116.36	172.50
Diastolic blood pressure (mmHg)	74.35	106.57
Proteinuria (mg/day)	–	769.19
Body weight of infant (g)	3243.29	2195.30
Gestational age (weeks)	39.45	35.87
Placental weight (g)	527.60	466.21

### Cell culture

Human placenta trophoblasts (HTR-8/SVneo, JEG-3, BeWo) and human embryonic kidney (HEK) 293 T cells were purchased from the American Type Culture Collection (ATCC, USA) and cultured in RPMI 1640 (Invitrogen, USA) with 10% fetal bovine serum (Invitrogen, USA) and 1% penicillin/streptomycin (Invitrogen, USA) at 37 °C in a humidified incubator with 5% CO_2_. In order to mimic the hypoxic condition in preeclampsia placenta, cells were cultured in a hypoxic workstation (AW200SG, Electrotek, UK) with 2% O_2_, 5% CO_2_ and 93% N_2_ at 37 °C. For the normoxic group, a mixture of 20% O_2_, 5% CO_2_ and 75% N_2_ was used instead. Culture medium was changed every two to three days. The treatment of HMGB1 was given as described in Son et al. (2016). Recombinant HMGB1 (ab167718, Abcam, USA) was added into the culture medium to a final concentration of 3 μg/mL, and cells were treated for 24 h.

### Cell transfection

The sh-LINC00240 vector for silencing of LINC00240 expression, the sh-Nrf2 vector for silencing of Nrf2 expression and the negative control sh-NC, the miR-155 inhibitor for silencing of miR-155 expression and the inhibitor NC, the miR-155 mimics and mimics NC were all designed and synthesized by GenePharma (Shanghai, China). The above shRNAs, miRNA inhibitor and mimics, and their corresponding negative controls were transfected into cells using the Lipofectamine 2000 transfection reagent (Thermo Fisher Scientific, USA) according to the manufacturer’s guidelines. For overexpression of LINC00240 (OE-LINC00240), cells were transfected with lentivirus (LV)-LINC00240 (LV5-EF1a-GFP/Puro vector, GenePharma, China). The lentiviral vectors were transfected into cells for 24 h at a multiplicity of infection of 50, followed by puromycin antibiotic selection (5 µg/mL) for 4 days.

### Dual luciferase reporter assay

Wild type or mutant 3′-untranslated region (UTR) of Nrf2 (Nrf2-WT/Nrf2-MUT) or LINC00240 (LINC00240-WT/LINC00240-MUT) was constructed into pmirGLO Dual-Luciferase miRNA Target Expression plasmid (Promega, USA). The reporter plasmids were co-transfected with miR-155 mimics or miR-155 inhibitor into 293 T cells using Lipofectamine 2000 (Thermo Fisher Scientific, USA). The firefly and renilla luciferase activities were sequentially measured after 48 h using Dual-Luciferase Reporter Assay System (Promega, USA) and the firefly luciferase results were normalized to renilla luciferase activities.

### RNA binding protein immunoprecipitation (RIP) assay

To demonstrate that LINC00240 could bind to miR-155, RIP assay was performed as described in Meier et al. (2013). Briefly, 293 T cells transfected with miR-155 mimics or mimics NC were lysed in polysome lysis buffer and stored at − 80 °C. Sepharose beads were coated with anti-Ago2 antibody (1:500, Sigma-Aldrich, USA) or control IgG antibody (1:500, Sigma-Aldrich, USA). After thawing, cell lysates were mixed at 1:10 with a cocktail buffer containing RNAseOUT (Invitrogen, USA) and then transferred into tubes containing beads coated with Ago2 or control IgG antibody. After incubation at 4 °C, beads were collected by centrifugation and washed in NT2 buffer. The proteins were eluted using glycine and then degraded by proteinase K (Sigma-Aldrich, USA). The immunoprecipitated RNAs were isolated using TRIzol reagent (Invitrogen, USA) for further measurement using quantitative PCR.

### Measurement of cell viability by CCK-8

An equal amount of cells (5 × 10^3^ cells per well) were transferred into wells of a 96-well plate with culture medium and incubated for indicated time. Cell Counting Kit-8 (CCK-8, #96992, Sigma-Aldrich, USA) was added into the cell culture medium at 1:10. After incubation at 37 °C for 2 h, absorbance was measured at 450 nm using a plate reader (Thermo Fisher Scientific, USA).

### Measurement of cell proliferation by colony formation assay

Cultured cells were firstly trypsinized and suspended in culture medium. Cells were diluted and planted in a 6-well plate at a density of 100 cells per well. After incubation for 2 weeks, colonies were fixed with 3.7% methanol and stained with 0.5% crystal violet at room temperature for 2 h. The number of colonies was counted under a microscope (Zeiss, Germany).

### Measurement of cell migration by wound healing assay

Cells were firstly seeded in 6-well culture plates (6 × 10^5^ cells per well) with culture medium. After cells reached 90% confluence, the plate was scratched across the center of the well using a 10 µL pipette tip. After the scratching, wells were gently washed with PBS to remove detached cells and image of the wound was taken under an inverted microscope (Zeiss, Germany). Culture medium was then added and cells were cultured for another 24 h. After washing with PBS, images of the wound were taken under microscope. Widths of the wound were measured using Image J software, and the migration ability of the cells was evaluated by the following formula: (W_0 h_ − W_24 h_)/W_0 h_ × 100%.

### Measurement of cell invasion by Transwell assay

Transwell assay was performed to study the cell invasion capacity using Transwell permeable supports (8 μm, Corning, USA). After indicated treatments, cells were harvested and diluted in serum-free medium. Cells (5 × 10^4^) were seeded in the upper chamber which was pre-coated with matrigel, whereas fresh medium containing 10% FBS was added to the bottom chamber. After further incubation for 48 h, cotton swab was used to remove the cells on the upper side of the filter. The invaded cells were fixed in 4% paraformaldehyde, and then stained with 1% crystal violet and counted under the microscope (Zeiss, Germany).

### Co-culture system and detection of M2 macrophage using flow cytometry

Human placenta trophoblast cell lines (HTR-8/SVneo, JEG-3), with or without transfection, were cultured in normoxic or hypoxic condition. After 48 h, supernatants of those cultured cells were taken and used to treat M0 macrophages for 24 h. After the treatment, flow cytometry was used to assess the phenotype of M2 macrophages (both positive in CD68 and Arg-1) as described in Lindau et al. (2018). Macrophages were resuspended in PBS and incubated with antibodies against CD68 (12-0689-42, Thermo Fisher Scientific) and Arg-1 (17-3697-82, Thermo Fisher Scientific) at 4 °C for 30 min in dark. After washing with PBS, cells were resuspended in PBS and analyzed using FACSCanto II (BD Biosciences, USA) with gating at CD68^+^Arg-1^+^. Results were obtained using FACSDiva 6.0 software (BD Biosciences, USA).

### Measurement of cell pyroptosis using flow cytometry

FAM-FLICA in vitro Caspase-1 Detection Kit (ImmunoChemistry, USA) was used to measure levels of caspase-1 following the manufacturer’s instruction. Briefly, each sample was added 5 μL FLICA and 2 μg/mL PI to a final volume of 300 μL. After incubation at 37 °C in dark for 1 h, cells were washed twice with wash buffer and analyzed with flow cytometer (BD Biosciences, USA).

### Assessment of oxidative stress

Reactive oxidative stress (ROS) Assay Kit (ab186027, Abcam, USA) and Lipid Peroxidation (MDA) Assay Kit (ab118970, Abcam, USA) were used to evaluate the oxidative stress levels as per the manufacturer’s instruction. For ROS assay, cultured cells were stained with ROS Red working solution and fluorescence intensity was measured at 520/605 nm (Ex/Em). For lipid peroxidation assay, 600 μL of thiobarbituric acid solution was added to 200 μL samples or standards. After incubation at 95 °C for 1 h, samples and standards were cooled to room temperature in ice bath. 200 μL of the reaction mixture from each sample or standard was moved into a 96-well plate and optical density (OD) value was immediately measured at 532 nm using a plate reader (Thermo Fisher Scientific, USA).

### ELISA assay

ELISA Kit for HMGB1 (ABIN6574155, Antibodies-online, Germany) was used to measure HMGB1 level in cell supernatant following the manufacturer’s instruction. Briefly, samples and standards were added into wells of a 96-well plate pre-coated with anti-HMGB1 antibody and incubated at 37 °C for 2 h. After discarding the supernatant and washing the wells with wash buffer, 100 μL of Detection Reagent A was added into the wells and the plate was incubated at 37 °C for 1 h. After washing, 100 μL of Detection Reagent B was added and the plate was incubated at 37 °C for 30 min. After complete washing of the wells, 90 μL of Substrate Solution was added and the plate was incubated at 37 °C for 15 min. 50 μL of Stop Solution was then added and OD value was measured at 450 nm using a plate reader (Thermo Fisher Scientific, USA).

### Animal model

Female Sprague–Dawley rats weighing from 200 to 250 g were obtained from the Shanghai SLAC Laboratory Animal Center (Shanghai, China) and kept in temperature and humidity-controlled environment with 12:12 h light–dark cycle. Animals were given free access to water and food and cages were changed three times per week. After acclimation for one week, the animals were mated overnight with healthy male rats. After confirming the pregnancy by presence of sperms in vagina (defined as embryonic day 0) (Li et al. 2018a; Xu et al. 2019, 2021), animals were randomly put into four groups: control group, lipopolysaccharide (LPS) group, LPS + OE-NC group and LPS + OE-LINC00240 group (n = 12 per group). For LPS group, 1.0 μg/kg LPS (Sigma-Aldrich, USA) was injected through tail vein on the 5th embryonic day to generate preeclampsia model. For control group, normal saline was injected instead of LPS on the same day. For LPS + OE-NC or LPS + OE-LINC00240 group, 1.0 μg/kg LPS was also injected through tail vein on the 5th embryonic day, and lentivirus vector carrying human LINC00240 by PCR or control vector was injected on the 6th and 15th embryonic day to generate LINC00240-overexpressed preeclampsia rat model. Animals were monitored for blood pressure and urine protein levels on embryonic day 0, 3, 6, and every 3 days thereafter. On embryonic day 20, animals were euthanized by cervical vertebra dislocation. Fetus, placenta, liver and kidney tissues were collected, weighed, and frozen at − 80 °C until further analyses. All the animal experiment procedures were approved by the Animal Ethics Committee of Henan Provincial People’s Hospital (Zhengzhou, Henan, China). All the animal experiments were also performed in Henan Provincial People’s Hospital.

### Immunohistochemistry assay

Rat placenta samples were collected and fixed in 4% paraformaldehyde for 24 h. After fixation, samples were dehydrated, embedded in paraffin, and stored at room temperature for further analyses. Before staining, the paraffin-embedded placenta samples were sectioned, and slices were de-paraffinized and hydrated in de-ionized water. After antigen-retrieval, slices were stained with anti-cytokeratin 7 (CK7) antibody (1:1000, ab199718, Abcam, USA) at 4 °C overnight, followed by HRP-conjugated goat anti-rabbit IgG secondary antibody (1:5000, ab205718, Abcam, USA) for 1 h. Slices were then counterstained by hematoxylin for 30 min. After washing, slices were visualized by 3,3′-diaminobenzidine, and staining results were examined under a light microscope (Zeiss, Germany).

### H&E staining assay

Paraffin-embedded rat kidney and liver samples were sectioned, followed by three changes of xylene and hydration in 100%, 95%, 80% ethanol. The slices were stained with hematoxylin for 3 min and rinsed three times with de-ionized water for 30 s, followed by differentiation in 1% acid ethanol. The slices were then stained with 0.5% eosin for 30 s and dehydrated in ethanol, followed by three changes of xylene. A drop of neutral balsam mounting medium was added. The slices were naturally dried overnight and observed under a microscope (Zeiss, Germany).

### Western blot assay

Cells were firstly lysed in RIPA buffer (Sigma-Aldrich, USA) and protein concentrations of each homogenate were measured using a commercial BCA protein assay Kit (Thermo Fisher Scientific, USA). 30 μg of proteins were separated on SDS-PAGE and transferred to a nitrocellulose membrane. The membrane was then blocked in 5% skimmed milk in TBST, and stained overnight at 4 °C with primary antibodies against Nrf2 (1:1000, ab137550, Abcam), HO-1 (1:2000, ab13243, Abcam), eNOS (1:5000, ab76198, Abcam), NQO1 (1:10,000, ab80588, Abcam), caspase-1 (1:1000, ab238979, Abcam), NLRP3 (1:1000, ab263899, Abcam), ASC (1:1000, ab180799, Abcam), HMGB1 (1:10,000, ab79823, Abcam), or β-actin (1:5000, ab115777, Abcam). Samples were then stained with goat anti-rabbit or goat anti-mouse IgG secondary antibody (1:10,000, ab205718/ab205719, Abcam) at room temperature for 2 h. Bands were developed using chemiluminescence substance (Thermo Fisher Scientific, USA). The proteins were quantified using Quantity One software (Bio-Rad, USA).

### Quantitative PCR (qPCR) assay

Total RNA from cells or tissue samples was extracted using TRIzol reagent (Invitrogen, USA). Reverse transcription was performed using 1 μg total RNA from each sample using PrimeScript RT reagent Kit (for mRNAs, Takara, China) or TaqMan MicroRNA Reverse Transcription Kit (for miR-155, Thermo Fisher Scientific, USA). qPCR was performed using SYBR Premix EX Taq Kit (Takara, China) on Applied Biosystems 7500 Real Time PCR system (Thermo Fisher Scientific, USA). β-actin and U6 small nuclear RNA (U6 snRNA) were used as internal references for mRNA and miRNA, respectively. Relative expression level was calculated by the 2^−ΔΔCt^ method. Specific primers for qPCR were obtained from Sangon Biotech (China) as listed in Table 2.

**Table 2:** Primers used for qRT-PCR

Primer	Direction	Primer sequence (5′–3′)
LINC00240	Forward	ATGCTCTAGGAGAAGCCAGC
	Reverse	AGTAGTTGAGGGTTGGCAAGG
MiR-155	Forward	GCGGGTTAATGCTAATCGTGATA
	Reverse	GTCGTATCCAGTGCAGGGTCCGAGGTATTCGCACTGGATACGAC AACCCC
Nrf2	Forward	TGAGCCCAGTATCAGCAACA
	Reverse	AGTGAAATGCCGGAGTCAGA
iNOS	Forward	CAGCATGTACCCTCGGTTCT
	Reverse	GGGGATCTGAATGTGCTGTT
Arg-1	Forward	CCAAGGTCTGTGGGAAAAGCA
	Reverse	TACAGGGAGTCACCCAGGAG
HO-1	Forward	GCTCAAAAAGATTGCCCAGA
	Reverse	GCTCTGGTCCTTGGTGTCAT
eNOS	Forward	ACCCTCACCGCTACAACATC
	Reverse	CTGGCCTTCTGCTCATTCTC
NLRP3	Forward	AAGGCCGACACCTTGATATG
	Reverse	CCGAATGTTACAGCCAGGAT
HMGB1	Forward	ATATGGCAAAAGCGGACAAG
	Reverse	GCAACATCACCAATGGACAG
U6	Forward	CTCGCTTCGGCAGCACA
	Reverse	AACGCTTCACGAATTTGCGT
β-actin	Forward	TGACGATGCCGTGCTCGATG
	Reverse	ACGGCTCCGGCATGTGCAAG

### Cell–cell fusion

The cell–cell fusion of BeWo cells was detected and fusion index was calculated as described in Zhang et al. (2020) with some modifications. Briefly, forskolin was used to induce the syncytialization of BeWo cells. After being cultured for 24 h on poly-l-lysine-coated cover glass, BeWo cells with indicated treatments were treated with 30 μM forskolin (Cell Signaling Technology, USA) for 48 h. After the treatment, the culture medium was discarded and 4% paraformaldehyde was added to fix the cells by incubation for 20 min at room temperature. 0.5% Triton X-100 in PBS was used to permeabilize the cells for 3–5 min at room temperature. Blocking buffer was added to block the cells for 1 h at room temperature. Specific primary antibody anti-E-cadherin (Cell Signaling Technology, USA) was added in the blocking buffer and incubated with cells overnight at 4 °C. Primary antibody was washed out using PBS for 3 times, followed by incubation of Alexa fluor-conjugated secondary antibody for 2 h at room temperature. Cells were washed three times again by PBS and then treated with DAPI-containing mounting media (Thermo Fisher Scientific, USA). The cells were captured using fluorescence microscope (Zeiss, Germany).

### Statistical analysis

All the experiments were repeated at least three times. Data were analyzed with GraphPad Prism 6.0 and results were expressed as mean ± standard deviation. Statistical evaluation was performed using Student’s t test between two groups or one-way analysis of variance (ANOVA) followed by Tukey’s post hoc test for multiple comparison. Spearman correlation analysis was performed to analyze the correlation between LINC00240, miR-155, Nrf2, Arg-1, and inducible nitric oxide synthase (iNOS) in placenta samples. *P* values less than 0.05 were considered as statistically significant for all analyses.

## Results

### LINC00240 is downregulated in placenta samples of preeclampsia patients

**Fig. 1:**
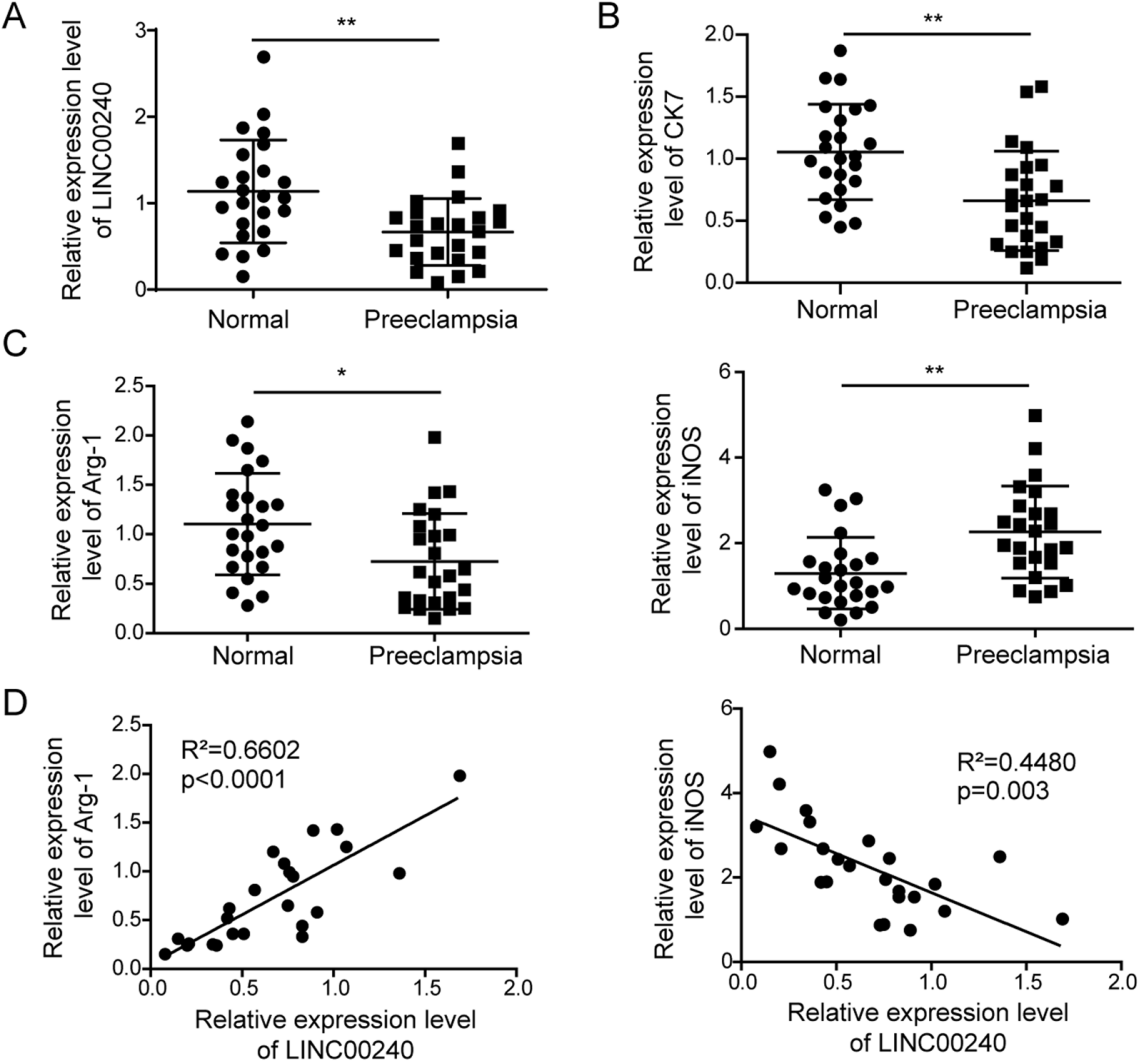
LINC00240 is downregulated in placenta samples of preeclampsia patients. **A** qPCR measurement on LINC00240 levels in placenta tissues from preeclampsia patients (n = 24) and normal pregnant women (n = 24). **B** qPCR measurement on CK7 levels in placenta tissues from preeclampsia patients (n = 24) and normal pregnant women (n = 24). **C** qPCR measurement on Arg-1 and iNOS levels in placenta tissues from preeclampsia patients (n = 24) and normal pregnant women (n = 24). **D** A correlation analysis between Arg-1, iNOS, and LINC00240 levels in placenta tissues from preeclampsia patients (n = 24). *P < 0.05 and **P < 0.01

In placenta samples from 24 clinically diagnosed preeclampsia patients, we found significantly lower LINC00240 (Fig. 1A) and CK7 (a trophoblast marker, Fig. 1B) expression compared to normal placenta samples. In addition, placenta samples of preeclampsia patients showed decreased levels of Arg-1 (a marker of M2 macrophage subtype) and increased levels of iNOS (a marker of M1 macrophage subtype) (Fig. 1C). Correlation analysis indicated a positive correlation between levels of LINC00240 and Arg-1, and a negative correlation between levels of LINC00240 and iNOS in placenta samples of preeclampsia patients (Fig. 1D). These results showed LINC00240 expression was related to macrophage polarization in placenta of preeclampsia.

### LINC00240 improves function of trophoblasts and M2 macrophage polarization in preeclampsia model in vitro

**Fig. 2:**
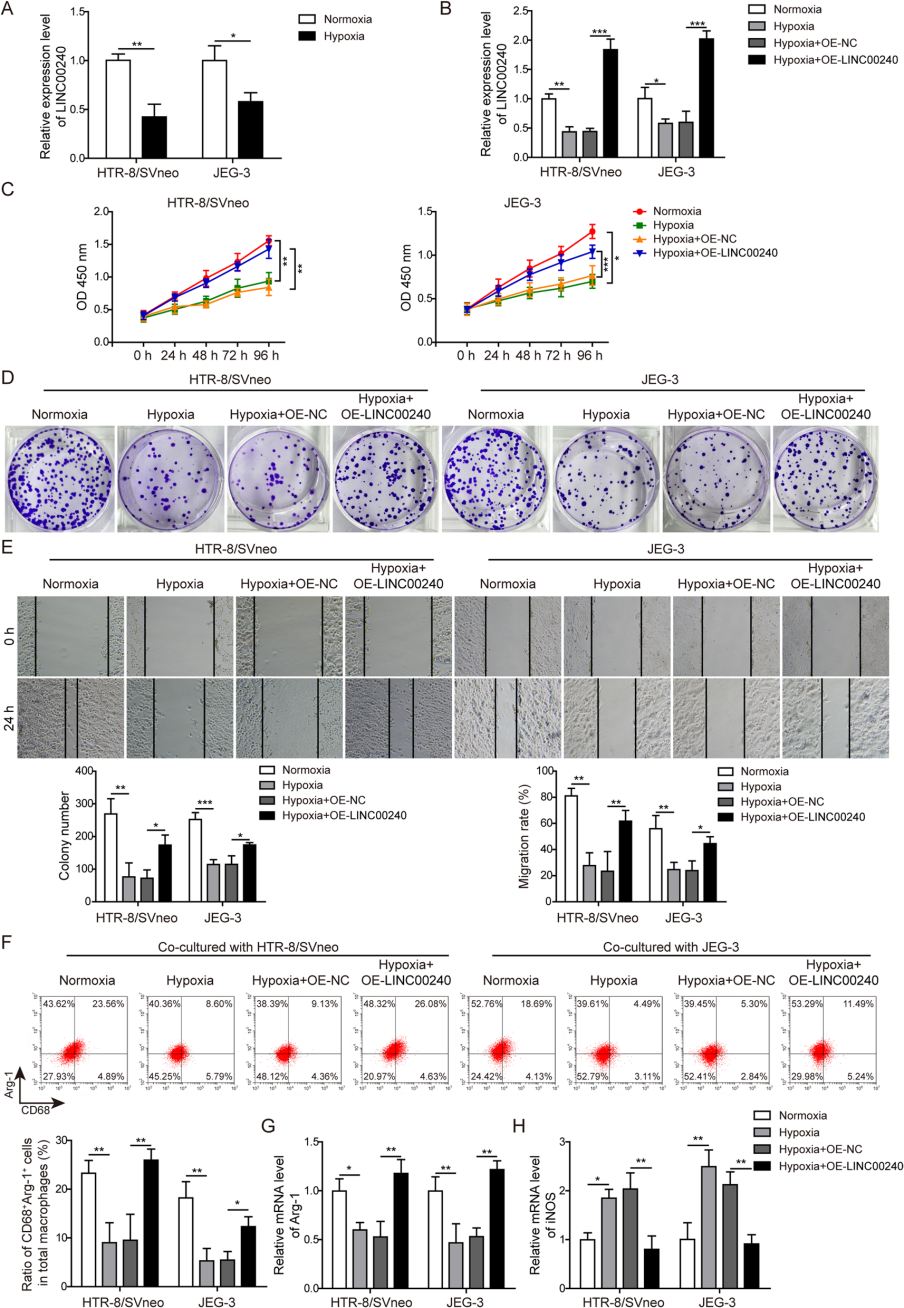
LINC00240 promotes function of trophoblasts and M2 macrophage polarization in preeclampsia model in vitro. **A** qPCR measurement on LINC00240 levels in two human trophoblasts (HTR-8/SVneo and JEG-3) cultured in a normal or hypoxic condition. **B** qPCR measurement on LINC00240 levels in trophoblasts under a normal or hypoxic condition with/without LINC00240 overexpression. **C** CCK-8 measurement on cell proliferation in indicated groups. **D** Colony formation assay on cell proliferation in indicated groups. **E** Wound healing assay on cell migration in indicated groups. **F** Flow cytometry analysis of M2 polarization markers (Arg-1, CD68) in macrophages co-cultured with trophoblasts after indicated treatments. **G** qPCR measurement on M2 marker (Arg-1) mRNA levels in macrophages co-cultured with trophoblasts after indicated treatments. **H** qPCR measurement on M1 marker (iNOS) mRNA levels in macrophages co-cultured with trophoblasts after indicated treatments. All the experiments were repeated at least three times. *P < 0.05, **P < 0.01 and ***P < 0.001

Similarly, human trophoblasts (HTR-8/SVneo and JEG-3) cultured in a hypoxic condition also showed lower LINC00240 expression (Fig. 2A). Overexpressing LINC00240 in HTR-8/SVneo and JEG-3 cells (Fig. 2B) showed increased proliferation (Fig. 2C, D) and migration (Fig. 2E) abilities of trophoblasts compared to cells cultured under hypoxic condition. Flow cytometry analysis revealed that macrophages presented less M2 subtypes (CD68^+^Arg-1^+^) when co-cultured with trophoblasts under a hypoxic condition (Fig. 2F), indicating the changes in macrophage polarization. When co-cultured with LINC00240-overexpressed trophoblasts, macrophages showed more M2 polarization compared to cells cultured in a hypoxic condition (Fig. 2F). These results were further convinced by observation of macrophage polarization markers that Arg-1 was increased while iNOS was decreased when co-cultured with LINC00240-overexpressed trophoblasts (Fig. 2G, H). These results indicated that LINC00240 was involved in the promotion of M2 macrophage polarization, as well as the function of trophoblasts in preeclampsia.

### LINC00240 negatively regulates miR-155 expression in trophoblasts

**Fig. 3:**
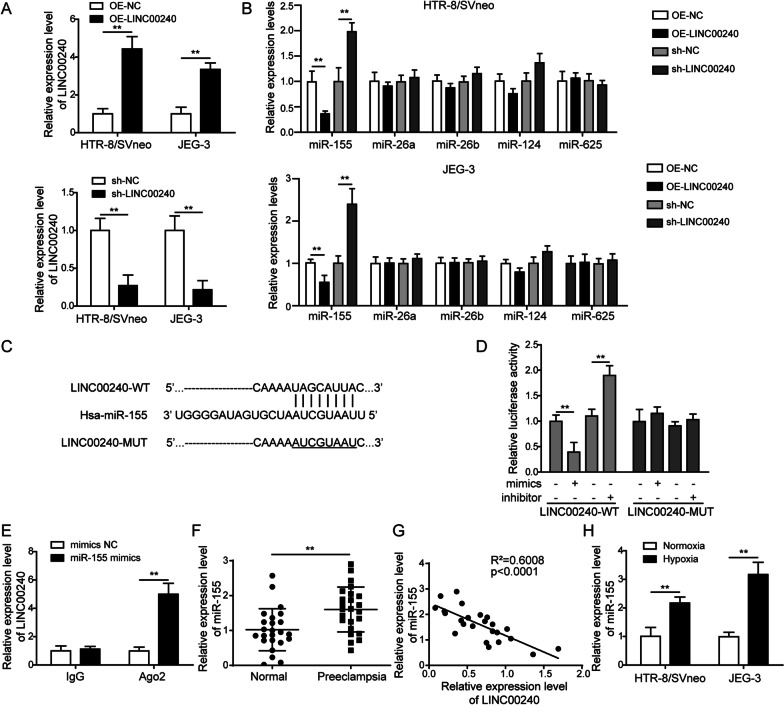
LINC00240 negatively regulates miR-155 expression in trophoblasts. **A** qPCR measurement on LINC00240 expression levels in HTR-8/SVneo and JEG-3 cells with LINC00240 overexpression (OE-LINC00240) or LINC00240 knockdown (sh-LINC00240). **B** qPCR measurement on miR-155, miR-26a, miR-26b, miR-124, and miR-625 expression levels in HTR-8/SVneo and JEG-3 cells with LINC00240 overexpression or knockdown. **C** Bioinformatics analysis of the possible binding sites between LINC00240 and miR-155. **D** Measurement of luciferase activities of wild type (WT) or mutated (MUT) LINC00240 using dual luciferase assay with enhanced or inhibited miR-155 expression. **E** Detection of direct binding between LINC00240 and miR-155 using RIP assay. **F** qPCR measurement on miR-155 expression levels in placenta tissues from preeclampsia patients (n = 24) and normal pregnant women (n = 24). **G** A correlation analysis between LINC00240 and miR-155 levels in placenta tissues from preeclampsia patients (n = 24). **H** qPCR measurement on miR-155 expression levels in HTR-8/SVneo and JEG-3 cells cultured in a normal or hypoxic condition. All the experiments were repeated at least three times. *P < 0.05 and **P < 0.01

In order to validate the regulation of LINC00240 on miRNAs, we overexpressed or knocked down LINC00240 in trophoblasts (Fig. 3A). Among the miRNAs (miR-155, miR-26a, miR-26b, miR-124, and miR-625) which were predicted to potentially bind to LINC00240 by bioinformatics analysis, changes in the expression level of miR-155 were the most significant when LINC00240 was overexpressed or knocked down, the decreased or increased miR-155 levels were found, respectively (Fig. 3B). Those results indicated an inhibitory regulation of LINC00240 on miR-155. Bioinformatics analysis revealed the potential binding sites between these two molecules (Fig. 3C). On the other hand, dual luciferase assay showed that miR-155 overexpression inhibited while miR-155 inhibition enhanced the luciferase activity in wild type of LINC00240 group, which was not observed in mutated LINC00240 group (Fig. 3D). RIP assay also showed significantly increased immunoprecipitation of LINC00240 in miR-155-overexpressed cells (Fig. 3E). Considering the inhibitory relationship between LINC00240 and miR-155 as observed in the above results, we further analyzed miR-155 levels in clinical samples, and elevated miR-155 expression levels were found in placenta of preeclampsia patients compared to normal placenta samples (Fig. 3F). Spearman correlation analysis revealed negative correlation between LINC00240 and miR-155 levels in clinical placenta samples of preeclampsia patients (Fig. 3G), further convincing inhibitory regulation between these two factors. Furthermore, miR-155 expression levels were elevated in trophoblasts under a hypoxic condition (Fig. 3H), suggesting that miR-155 might also play important roles in the pathogenesis of preeclampsia. The above findings indicated that LINC00240 negatively regulated the expression of miR-155 in preeclampsia.

### LINC00240 inhibits oxidative stress-induced pyroptosis of trophoblasts by negatively targeting miR-155 in preeclampsia model in vitro

**Fig. 4:**
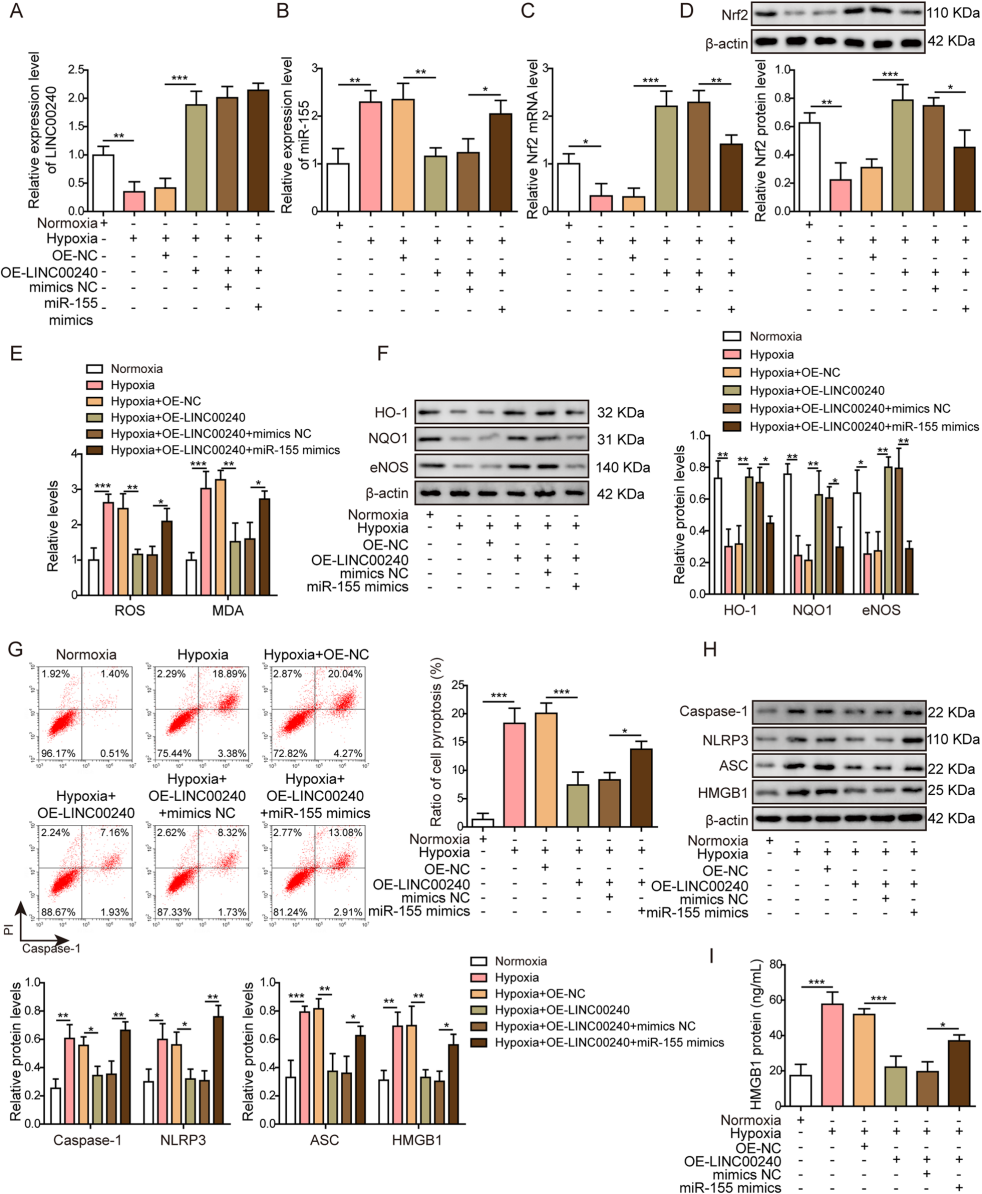
LINC00240 inhibits oxidative stress-induced pyroptosis of trophoblasts in preeclampsia model in vitro by negatively targeting miR-155. **A** LINC00240 levels were measured using qPCR in HTR-8/SVneo cells after indicated treatments. **B** MiR-155 levels were measured using qPCR in HTR-8/SVneo cells after indicated treatments. **C** Nrf2 mRNA levels were measured using qPCR in HTR-8/SVneo cells after indicated treatments. **D** Nrf2 protein levels were measured by western blotting in HTR-8/SVneo cells after indicated treatments. **E** ROS and MDA levels were measured using ROS assay Kit and MDA assay Kit, respectively. **F** Protein levels of HO-1, NQO1 and eNOS were measured by western blotting. **G** Caspase-1, a pyroptosis marker, was measured using flow cytometry. **H** Protein levels of pyroptosis markers (caspase-1, NLRP3, ASC and HMGB1) were measured by western blotting. **I** HMGB1 protein levels were measured using ELISA. All the experiments were repeated at least three times. *P < 0.05, **P < 0.01 and ***P < 0.001

To further validate the regulatory relationship between LINC00240 and miR-155 and their regulation on cell pyroptosis, we generated preeclampsia model in vitro with both LINC00240 and miR-155 overexpression, and firstly measured LINC00240, miR-155, and Nrf2 expression levels. The results showed that the hypoxic condition led to decreased LINC00240 and Nrf2 expression levels and elevated miR-155 expression, which were reversed by LINC00240 overexpression (Fig. 4A–D). Further overexpression of miR-155 in trophoblasts only reversed miR-155 and Nrf2 expression levels caused by LINC00240 overexpression (Fig. 4A–D), suggesting that miR-155 is a downstream signaling factor of LINC00240.

Measurement of oxidative stress biomarkers indicated that hypoxia increased ROS and MDA levels (Fig. 4E) and decreased expression levels of HO-1, NQO1, and eNOS (Fig. 4F), indicating increased oxidative stress in trophoblasts. Overexpression of LINC00240 inhibited the effects of hypoxia on the above oxidative stress markers in trophoblasts, while further overexpression of miR-155 reversed the inhibitory effects of LINC00240 on oxidative stress (Fig. 4E, F). Since Nrf2/HO-1-mediated oxidative stress could regulate cell pyroptosis (Diao et al. 2019; Zhang et al. 2020b), we further investigated the possible regulation of LINC00240/miR-155 axis on cell pyroptosis. Flow cytometry analysis indicated that LINC00240 inhibited the ratio of cell pyroptosis induced by hypoxia, but miR-155 overexpression decreased the effects of LINC00240 (Fig. 4G). Investigation on cell pyroptosis markers demonstrated that hypoxia increased the protein levels of caspase-1, NLRP3, ASC, and HMGB1 compared to normal culture condition. Overexpression of LINC00240 decreased the protein levels of those pyroptosis markers, and further overexpression of miR-155 in those cells reversed the effects of overexpression of LINC00240 (Fig. 4H). ELISA assay also proved that hypoxia induced the secretion of HMGB1 in trophoblasts, while overexpression of LINC00240 inhibited the HMGB1 secretion. In addition, co-overexpression of miR-155 suppressed the effects of LINC00240 overexpression and increased the secretion of HMGB1 again (Fig. 4I). Those results suggested that LINC00240 could suppress oxidative stress-induced pyroptosis of trophoblasts by negatively targeting miR-155.

### LINC00240 improves M2 macrophage polarization and function of trophoblasts by negatively targeting miR-155 in preeclampsia model in vitro

**Fig. 5:**
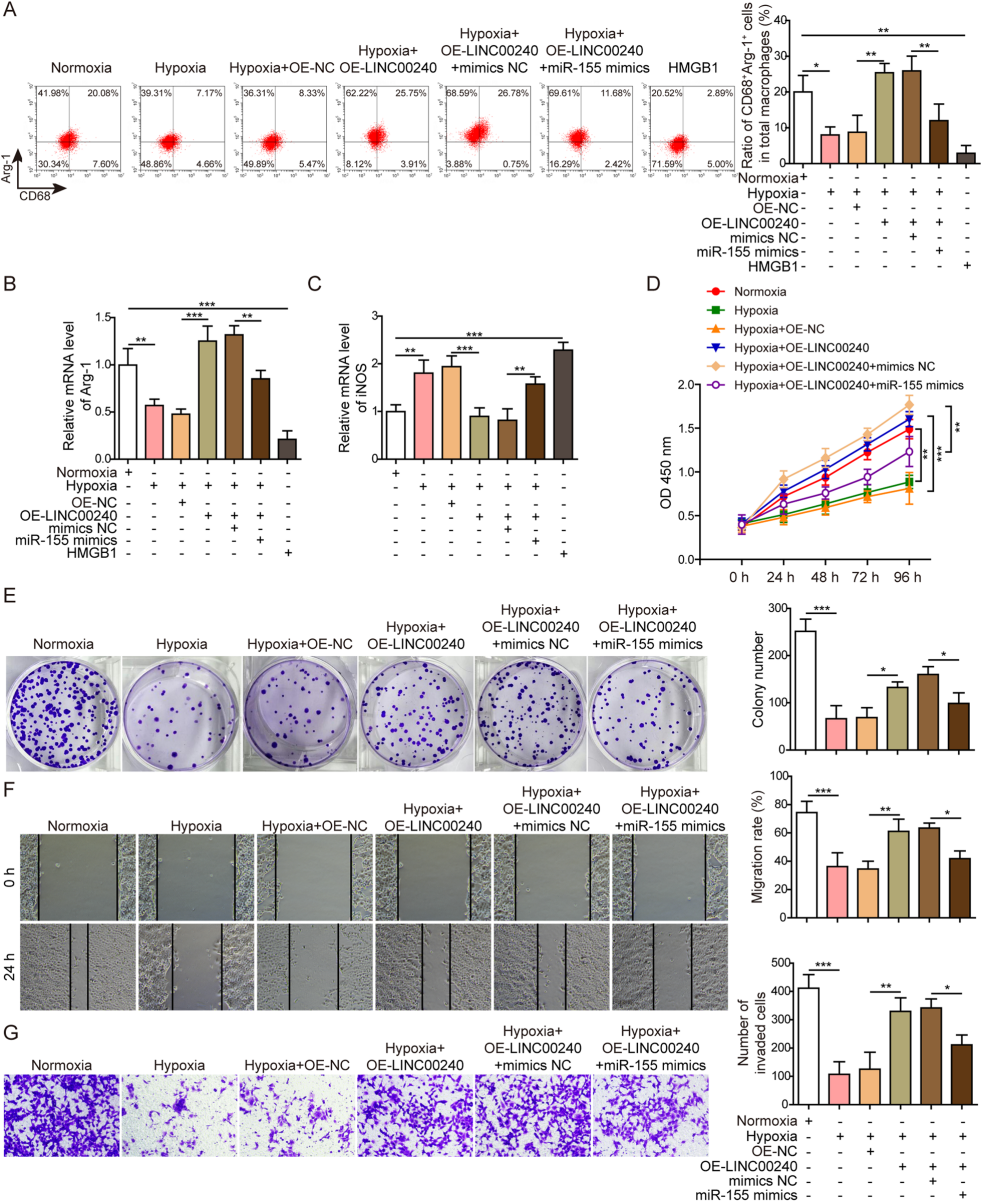
LINC00240 improves function of trophoblasts and M2 macrophage polarization in preeclampsia model in vitro by negatively targeting miR-155. **A** M2 macrophage marker (Arg-1, CD68) levels were measured by flow cytometry in macrophages co-cultured with trophoblasts after indicated treatments. **B** Arg-1 expression levels were measured by qPCR in macrophages co-cultured with trophoblasts after indicated treatments. **C** iNOS expression levels were measured by qPCR in macrophages co-cultured with trophoblasts after indicated treatments. **D** Cell proliferation was measured by CCK-8 assay in trophoblasts after indicated treatments. **E** Cell proliferation was measured by colony formation assay in trophoblasts after indicated treatments. **F** Cell migration ability was assessed using wound healing assay in trophoblasts after indicated treatments. **G** Cell invasion ability was measured using Transwell assay in trophoblasts after indicated treatments. All the experiments were repeated at least three times. *P < 0.05, **P < 0.01 and ***P < 0.001

We further investigated the macrophage polarization when co-cultured with trophoblasts under different conditions. Flow cytometry results showed increased M2 polarization of macrophages co-cultured with LINC00240-overexpressed trophoblasts compared to cells cultured in hypoxia, which was reversed by further overexpression of miR-155 (Fig. 5A). qPCR measurement on macrophage polarization markers showed similar results that M2 marker (Arg-1) was increased by LINC00240 overexpression, which was inhibited by miR-155 overexpression, while M1 marker (iNOS) level showed the opposite trend (Fig. 5B, C). Those results showed that LINC00240 induced M2 macrophage polarization by negatively targeting miR-155 in preeclampsia model in vitro.

Further investigation using CCK-8, colony formation, wound healing, Transwell and cell–cell fusion assays indicated that LINC00240 overexpression could increase the cell proliferation (Fig. 5D, E), migration (Fig. 5F), invasion (Fig. 5G), and fusion (Additional file 1: Fig. S1A, B) abilities of trophoblasts cultured in hypoxic condition, and these effects could be partially inhibited by miR-155 overexpression, indicating that LINC00240 improved the function of trophoblasts by negatively targeting miR-155 in preeclampsia model in vitro.

### MiR-155 negatively regulates Nrf2 expression in trophoblasts

**Fig. 6:**
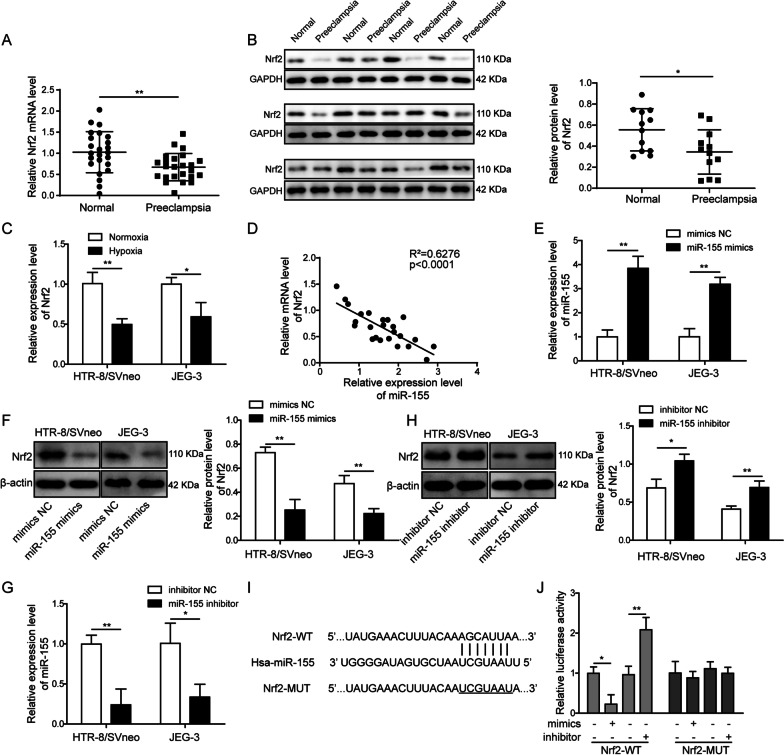
MiR-155 negatively regulates Nrf2 expression in trophoblasts. **A** qPCR measurement of Nrf2 mRNA levels in placenta tissues from preeclampsia patients (n = 24) and normal pregnant women (n = 24). **B** Western blot analysis of Nrf2 protein expression in placenta tissues from preeclampsia patients and normal pregnant women (n = 12, randomly selected from 24 placenta tissues from preeclampsia patients and 24 placenta tissues from normal pregnant women). **C** qPCR measurement of Nrf2 mRNA levels in HTR-8/SVneo and JEG-3 cells cultured under a normal or hypoxic condition. **D** A correlation analysis between miR-155 and Nrf2 mRNA levels in placenta tissues from preeclampsia patients (n = 24). **E** qPCR measurement of miR-155 levels in HTR-8/SVneo and JEG-3 cells transfected with miR-155 mimics or mimics NC. **F** Western blot analysis of Nrf2 protein expression in HTR-8/SVneo and JEG-3 cells transfected with miR-155 mimics or mimics NC. **G** qPCR measurement of miR-155 levels in HTR-8/SVneo and JEG-3 cells transfected with miR-155 inhibitor or inhibitor NC. **H** Western blot analysis of Nrf2 protein expression in HTR-8/SVneo and JEG-3 cells transfected with miR-155 inhibitor or inhibitor NC. **I** Bioinformatics analysis of the binding sites between miR-155 and Nrf2. **J** Measurement of luciferase activities of wild type (WT) or mutated (MUT) Nrf2 using dual luciferase assay with enhanced or inhibited miR-155 expression. All the experiments were repeated at least three times. *P < 0.05 and **P < 0.01

Nrf2 mRNA expression levels were also measured in the placenta tissues from preeclampsia patients and normal pregnant women, and results indicated decreased mRNA levels of Nrf2 in preeclampsia group (Fig. 6A). In addition, Nrf2 protein expression levels were also measured in placenta tissues from randomly-selected 12 preeclampsia patients and 12 normal pregnant women. The results also showed overall lower Nrf2 protein expression in preeclampsia group (Fig. 6B). Similar results were observed in trophoblasts cultured in a hypoxic condition compared to a normal condition (Fig. 6C). Correlation analysis showed a negative correlation between miR-155 and Nrf2 levels in placenta tissues from preeclampsia patients (Fig. 6D). Furthermore, after overexpressing miR-155 in trophoblasts (Fig. 6E), the expression level of Nrf2 was inhibited (Fig. 6F). However, inhibition of miR-155 expression led to increased Nrf2 expression (Fig. 6G, H). Bioinformatics analysis also indicated potential binding sites between miR-155 and Nrf2 (Fig. 6I). The relative luciferase activity was decreased when miR-155 expression was enhanced, and increased when miR-155 expression was inhibited in Nrf2 wild type group, while no significant changes were observed in mutated Nrf2 group (Fig. 6J). These results suggested miR-155 negatively regulated Nrf2 expression in placenta trophoblasts.

### Silencing of miR-155 suppresses oxidative stress-induced pyroptosis of trophoblasts by upregulating Nrf2 in preeclampsia model in vitro

To further demonstrate the roles of miR-155/Nrf2 axis on cell pyroptosis in preeclampsia, we inhibited both miR-155 and Nrf2 expression in trophoblasts cultured in a hypoxic condition. Hypoxia increased expression level of miR-155, which was reversed by direct inhibition of miR-155, but not by inhibition of Nrf2 (Additional file 2: Fig. S2A). Nrf2 expression level was increased after miR-155 inhibition as shown in both mRNA and protein levels, while further inhibition of Nrf2 led to decreased Nrf2 expression (Additional file 2: Fig. S2B, C). Further investigation on oxidative stress showed that miR-155 inhibition decreased ROS and MDA levels (Additional file 2: Fig. S2D) and increased protein levels of HO-1, NQO1, and eNOS (Additional file 2: Fig. S2E) induced by a hypoxic condition. Further inhibition of Nrf2 increased ROS and MDA levels, and decreased protein levels of HO-1, NQO1, and eNOS, compared to trophoblasts treated with a hypoxic condition and miR-155 inhibitor (Additional file 2: Fig. S2D, E). In a word, silencing of miR-155 inhibited oxidative stress of trophoblasts by up-regulating Nrf2 in preeclampsia model in vitro.

Similarly, flow cytometry results showed that miR-155 inhibition in trophoblasts resulted in a decreased ratio of cell pyroptosis in a hypoxic condition (Additional file 2: Fig. S2F), which was convinced by the decreased protein levels of caspase-1, NLRP3, ASC, and HMGB1 measured by western blotting (Additional file 2: Fig. S2G) or ELISA (Additional file 2: Fig. S2H). Further inhibition of Nrf2 also reversed the effects of miR-155 inhibition on cell pyroptosis (Additional file 2: Fig. S2F–H). All those results further demonstrated that inhibition of miR-155 could suppress oxidative stress-induced pyroptosis of trophoblasts through up-regulation of Nrf2.

### Silencing of miR-155 induces M2 macrophage polarization and function of trophoblasts in preeclampsia model in vitro by upregulating Nrf2

MiR-155 inhibition in trophoblasts induced the increased M2 macrophage polarization (CD68^+^Arg-1^+^) compared to cells cultured in a hypoxic condition (Additional file 3: Fig. S3A). qPCR results showed increased Arg-1 (M2 marker) and decreased iNOS (M1 marker) when miR-155 expression was inhibited (Additional file 3: Fig. S3B-C), and further inhibition of Nrf2 alleviated the effects of miR-155 inhibition on the polarization of co-cultured macrophages (Additional file 3: Fig. S3A–C). What’s more, inhibition on miR-155 expression increased the proliferation (Additional file 3: Fig. S3D, E), migration (Additional file 3: Fig. S3F), invasion (Additional file 3: Fig. S3G), and fusion (Additional file 4: Fig. S4A, B) abilities of trophoblasts compared to cells cultured in a hypoxic condition, while further inhibition on Nrf2 expression reversed the effects of miR-155 inhibition. Those results suggested that silencing of miR-155 could promote function of trophoblasts and M2 polarization of macrophages via up-regulation of Nrf2.

### LINC00240 overexpression ameliorates symptoms of preeclampsia in vivo

**Fig. 7:**
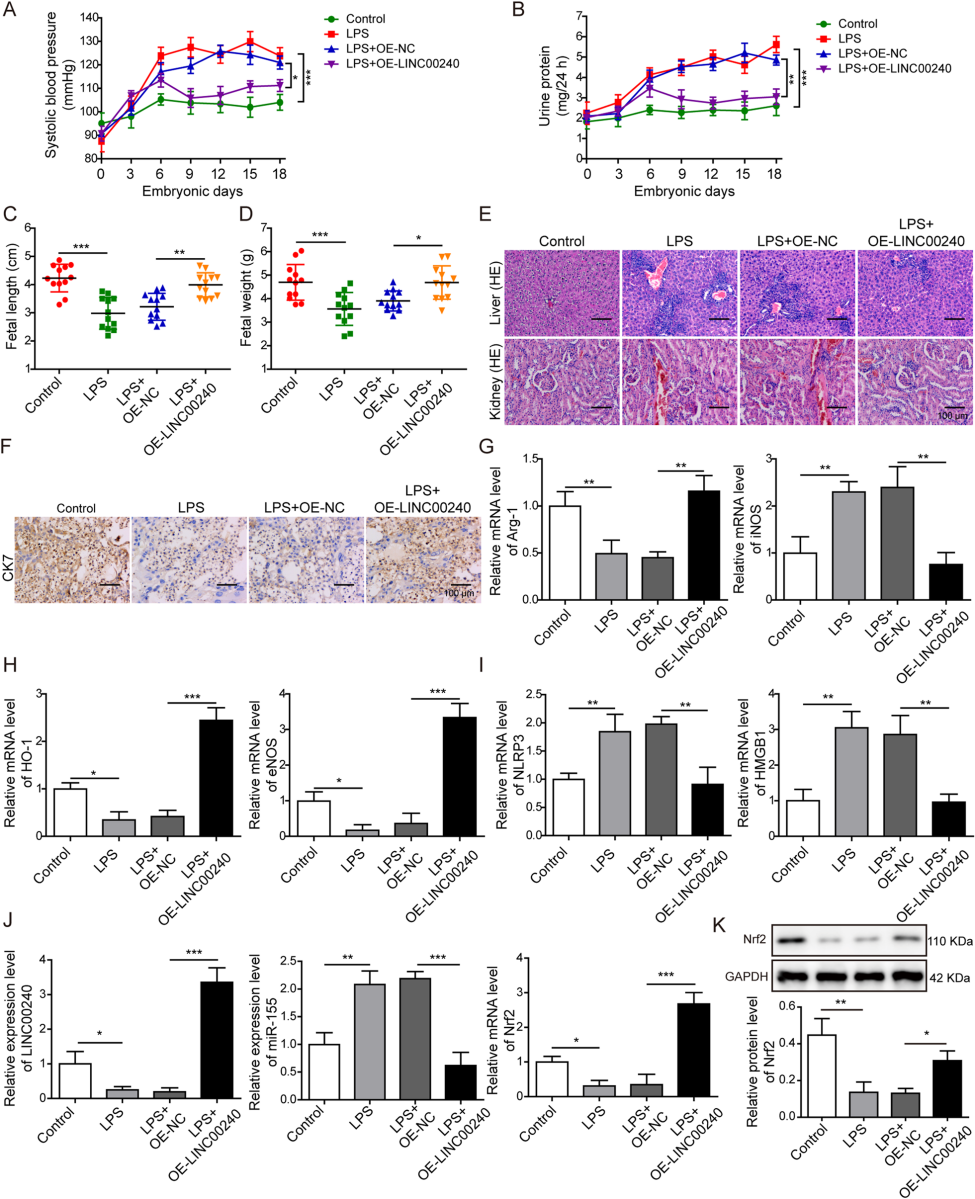
LINC00240 overexpression ameliorates symptoms of preeclampsia in vivo. **A** Blood pressure of rats was monitored on embryonic day 0, 3, 6 and every 3 days thereafter. **B** Urine protein levels were monitored on embryonic day 0, 3, 6 and every 3 days thereafter. **C** Fetal lengths of rats were measured on embryonic day 20. **D** Fetal weights of rats were measured on embryonic day 20. **E** Kidney and liver injuries were measured using H&E staining. Scale bar: 100 μm. **F** CK7 expression was examined using immunohistochemistry in placenta tissues of rats. Scale bar: 100 μm. **G** Arg-1 and iNOS mRNA levels in placenta tissues were measured using qPCR. **H** HO-1 and eNOS mRNA levels in placenta tissues were measured using qPCR. **I** NLRP3 and HMGB1 mRNA levels in placenta tissues were measured using qPCR. **J** LINC00240, miR-155 and Nrf2 levels in placenta tissues were measured using qPCR. **K** Nrf2 protein levels in placenta tissues were measured using western blotting. All the experiments were repeated at least three times. *P < 0.05, **P < 0.01 and ***P < 0.001

We generated a preeclampsia rat model by LPS injection on embryonic day 5. Monitoring on blood pressure and urine protein of rats showed elevated systolic blood pressure and urine protein levels in preeclampsia group, compared to control group (Fig. 7A, B). The length and weight of fetus were also lower in preeclampsia group (Fig. 7C, D). Overexpression of LINC00240 in the preeclampsia rat model significantly inhibited the elevation of blood pressure and urine protein levels (Fig. 7A, B) and elevated fetal length and weight (Fig. 7C, D). Kidney and liver damages were observed in the preeclampsia group compared to the control group, while overexpression of LINC00240 led to less kidney and liver damages compared to the preeclampsia group (Fig. 7E). Immunohistochemistry results revealed that the expression level of CK7, a trophoblast marker, was decreased in placenta tissues from the preeclampsia group, which was also reversed by LINC00240 overexpression (Fig. 7F).

Further investigation on molecular markers were consistent with our in vitro findings, Arg-1 level was decreased and iNOS level was increased in placenta tissues from the preeclampsia group (Fig. 7G), indicating the inhibited M2 polarization of macrophages. HO-1 and eNOS levels were decreased, and NLRP3 and HMGB1 levels were increased in the preeclampsia model (Fig. 7H, I), resulting in the elevation of oxidative stress and pyroptosis in the preeclampsia model. Expectedly, LINC00240 overexpression reversed the above effects on M2 polarization and oxidative stress-induced pyroptosis caused by LPS (Fig. 7G–I). Decreased LINC00240 and Nrf2 levels and increased miR-155 level were also observed in placenta tissues from the preeclampsia model, and further overexpression of LINC00240 reversed all the changes on LINC00240, miR-155 and Nrf2 expression levels (Fig. 7J, K). These results indicated that LINC00240 overexpression inhibited the oxidative stress-induced pyroptosis and promoted the M2 macrophage polarization in placenta tissues of preeclampsia.

## Discussion

Previous investigations revealed that the pathogenesis of preeclampsia involved hypoxia, increased oxidative stress and inflammation in placenta trophoblasts and M1 polarization of placenta macrophages (Hod et al. 2015; Vishnyakova et al. 2019). On the basis of these findings, our results for the first time indicated that expression of LINC00240 was inhibited in placenta tissues of preeclampsia patients and animals, and trophoblasts cultured in a hypoxic condition. Further investigations on the underlying mechanism revealed that up-regulation of LINC00240 increased the expression levels of Nrf2 through inhibition on miR-155 in trophoblasts cultured in a hypoxic condition. The overexpression of LINC00240 also inhibited the elevation of oxidative stress-induced pyroptosis of trophoblasts induced by hypoxia, thus improved the function of trophoblasts as well as promoted M2 macrophage polarization. Several methods such as injection of Nu-nitro-l-arginine-methyl ester (L-NA) (Ding et al. 2014, 2015; Huai et al. 2018) or LPS (Li et al. 2018a, b; Huai et al. 2018, 2020; Fan et al. 2019; Xue et al. 2020; Wu and Xiao 2019; Gong et al. 2016; Park et al. 2020), were used for the establishment of the animal model of preeclampsia. In our study, we used LPS to generate the in vivo model, and overexpression of LINC00240 in the in vivo preeclampsia model inhibited the symptoms of preeclampsia including hypertension, proteinuria, and damages to liver and kidney, making LINC00240 a potential therapeutic target for preeclampsia.

LINC00240 is a newly-identified lncRNA which was only studied in a few researches. Zheng et al*.* used the Subpathway-LNCE method to analyze the lncRNAs regulating the function of ischemic stroke, and LINC00240 was identified as one of the eleven hub-lncRNAs in this disease (Zheng et al. 2019). In addition, Yang et al*.* analyzed expression of mRNAs and lncRNAs in ESCC patients using bioinformatics methods, and the researchers identified two intrinsic subtypes of ESCC. The “loss” of crosstalk between LINC00240 and LOX gene family members was found in majority (94.5%) of subtype 2 of ESCC (Yang et al. 2016). Pathogenesis of preeclampsia was associated with dysfunction of placenta trophoblasts and the immune system under hypoxia, which was characterized by impaired trophoblast migration and invasion abilities, and abnormal polarization of macrophages (increased M1 subtype and decreased M2 subtype) in placenta (Vishnyakova et al. 2019; Przybyl et al. 2016; Li et al. 2016a; Buckley et al. 2016; Kolben et al. 2018). HTR-8/SVneo and JEG-3 cell lines have been widely used as in vitro models of trophoblasts in preeclampsia (Xu et al. 2019; Zhang et al. 2020c; Zhou et al. 2020; Abbas et al. 2020; Oh et al. 2020; Zeng et al. 2020; Zheng et al. 2020a), and many studies investigating preeclampsia were based on the proliferation, migration, and invasion of trophoblasts (Xu et al. 2019; Xue et al. 2020; Zhou et al. 2020; Abbas et al. 2020; Oh et al. 2020; Zeng et al. 2020; Zhang et al. 2019; Jiang et al. 2020; Zheng et al. 2020b). Our study is the first to report roles of LINC00240 in preeclampsia using these in vitro trophoblast models (HTR-8/SVneo and JEG-3). In our results, decreased expression of LINC00240 was shown in placenta tissues of preeclampsia patients and hypoxia-induced in vitro preeclampsia cell model. Overexpression of LINC00240 in in vitro preeclampsia cell model and in vivo preeclampsia rat model promoted proliferation, migration, invasion, and fusion of placenta trophoblasts and M2 polarization of placenta macrophages. Although increase of trophoblast fusion may promote preeclampsia through cellular senescence and earlier placental aging (Cox and Redman 2017), the overexpression of LINC00240 exerted protective effects on trophoblasts under a hypoxic condition. Also, overexpression of LINC00240 significantly inhibited hypertension, proteinuria, and liver and kidney damages, and promoted the growth of fetus in the in vivo preeclampsia rat model. This is the first study to explore the roles of LINC00240 on modulating function of trophoblasts and M2 macrophage polarization in preeclampsia. In addition, our study is the first to report that LINC00240 relieved the preeclampsia in an in vivo disease model. Our findings indicated that LINC00240 could be used as a potential clinical therapeutic target for preeclampsia, and possibly be able to relieve placenta damages and clinical manifestations of this disease.

Pyroptosis was also seldom studied in preeclampsia. Duan et al*.* reported that cell pyroptosis of trophoblasts was activated in preeclampsia which was inhibited by tumor necrosis factor-related protein 4 (Duan et al. 2016). Cheng et al*.* showed that pyroptosis was induced in trophoblasts of preeclampsia patients, and placental pyroptosis could be a key event in the pathogenesis of preeclampsia (Cheng et al. 2019). Pyroptosis is an inflammatory type of programmed cell death triggered by increased oxidative stress (Miao et al. 2011), which is involved in cell death and inflammatory reaction in cardiovascular diseases (Zhaolin et al. 2019) and could influence the proliferation and invasion of tumor cells (Fang et al. 2020). Similarly, our study also found an elevation of oxidative stress-induced pyroptosis in both in vitro and in vivo preeclampsia models, which inhibited the proliferation, migration, and invasion of trophoblasts, and M2 polarization via secretion of HMGB1. Furthermore, to the best of our knowledge, our study is the first to report that LINC00240 could inhibit oxidative stress-induced pyroptosis of trophoblasts.

Accumulating evidence suggested key roles of miRNAs in the pregnancy-related complications, such as preeclampsia (Lagana et al. 2018) and fetal growth restriction (Chiofalo et al. 2017). As a type of miRNA, roles of miR-155 in preeclampsia were investigated in several previous studies, and were reported to be elevated in preeclampsia (Azizi et al. 2017; Jairajpuri et al. 2017; Gan et al. 2017) and regulate eNOS, cyclin D1, and CYR61 expression levels in placenta (Li et al. 2014; Zhang et al. 2010; Dai et al. 2012; Kim et al. 2017). Our investigation also found increased miR-155 expression in placenta tissues of preeclampsia patients and hypoxia-induced preeclampsia cell model. In addition, miR-155 expression was reversely correlated with LINC00240 in preeclampsia placenta tissues. Furthermore, our bioinformatics results also showed that LINC00240 could directly bind to miR-155, and luciferase reporter assay and RIP assay showed that LINC00240 could inhibit miR-155 expression. Suppression of miR-155 inhibited oxidative stress-induced pyroptosis in trophoblasts, promoting trophoblast cell proliferation, migration, invasion, and fusion, and M2 polarization of placenta macrophages. Previous studies reported that miRNAs, including miR-155, were key regulators of macrophage polarization in atherosclerosis and other inflammation-related diseases (Bruen et al. 2019; Essandoh et al. 2016). In addition, miR-155 was reported to promote pyroptosis in macrophages (Li et al. 2019) and cardiomyocytes (Wang et al. 2020a). To the best of our knowledge, our study is the first to report that LINC00240 could directly target and regulate the expression of miR-155 to modulate function of trophoblasts and M2 polarization.

Nrf2 was reported to be a downstream factor of miR-155 in lung cancer and liver injury (Gu et al. 2017; Yang et al. 2018). Nrf2 was also reported to be involved in the regulation of oxidative stress through HO-1, and Nrf2/HO-1 could also prevent pyroptosis by modulating calcium level (Zhang et al. 2020b; Ma 2013; Sajadimajd and Khazaei 2018). Our study showed that Nrf2 expression levels were decreased in placenta tissues of preeclampsia patients, which were reversely correlated with miR-155 levels. MiR-155 was also shown to directly regulate Nrf2 in placenta trophoblasts. Inhibition of miR-155 could up-regulate Nrf2 expression to inhibit oxidative stress-induced pyroptosis of trophoblasts and then promote trophoblast cell proliferation, migration, invasion, and fusion, and M2 macrophage polarization. Taken together, the above results indicated that miR-155/Nrf2 axis was also one of the key changes in pathogenesis of preeclampsia, and our study is the first to investigate the regulatory relationship between miR-155 and Nrf2 in preeclampsia.

In a word, in the era of molecular medicine, identification of proper biomarkers is both important and challenging for the early diagnosis of preeclampsia (Lagana et al. 2016, 2017). Our study showed that overexpression of LINC00240 suppressed oxidative stress-induced pyroptosis to improve the trophoblast function and polarization of M2 macrophages through regulation on miR-155/Nrf2 axis. To the best of our knowledge, our study is the first to report the roles of LINC00240 in preeclampsia and the regulatory relationship between LINC00240, miR-155, and Nrf2 in this disease. In addition, our study results suggested that LINC00240 could be used as a potential therapeutic target to relieve the symptoms of preeclampsia. The limitation of this study could be the relatively small amount of clinical samples used, and that all clinical samples were from China, which may partially explain the relatively small difference found in the LINC00240 expression between placenta tissues from preeclampsia patients and normal pregnant women. Other limitation of this study could be the different species for in vitro disease model (human cell lines) which were widely used in studying preeclampsia (Qu et al. 2018; Li et al. 2018c; Fang et al. 2018; Cao et al. 2017) and in vivo disease model (rat). Due to the ethical issues, it was difficult for us to use the same species in the in vivo model as in vitro model. Therefore, we chose rat species as the in vivo disease model, which was commonly practiced in many previous studies that also both used the human and rat species (Xu et al. 2019; Fan et al. 2019; Xue et al. 2020; Peng et al. 2020; Wang et al. 2020b). Future investigations using the same species in both in vitro and in vivo models may be required to better support our hypothesis. In addition, the in vitro and in vivo preeclampsia models generated by hypoxia or LPS injection in our study may not fully mimic the preeclampsia in human patients since the LPS induces hypertension even outside gestation (Steib et al. 2010) and preeclampsia develops even without previous signs of hypertension. Clinical investigations are required to further validate the hypothesis.

### Clinical perspectives

Preeclampsia could lead to many manifestations in both mother and fetus, and may cause serious symptoms such as stroke, seizure, and even death. It is therefore of great clinical importance to investigate the roles of trophoblast function and macrophage polarization in the treatment of preeclampsia.

LINC00240 could regulate the expression of miR-155, which then modulated Nrf2 expression and inhibited oxidative stress-induced pyroptosis. The inhibited pyroptosis on one hand promoted proliferation, migration, invasion and fusion of trophoblasts, and on the other hand promoted M2 and inhibited M1 polarization of macrophages in preeclampsia through decreasing secretion of HMGB1.

On the basis of previous findings, our studies further shed light on the underlying molecular mechanism of preeclampsia. To the best of our knowledge, our study is the first to show the roles of LINC00240 in preeclampsia. LINC00240 could be a potential therapeutic target for preeclampsia.

## Supplementary Information

**Additional file 1: Fig. S1.** LINC00240 improves fusion ability of BeWo cells by negatively targeting miR-155 in preeclampsia model in *vitro*. After being cultured for 24 h on poly-L-lysine-coated cover glass, BeWo cells with indicated treatments were treated by 30 μM forskolin for 48 h to induce the syncytialization of BeWo cells. Immunofluorescence assay was used to visualize the cell membrane and nucleus with anti-E-cadherin antibody and DAPI, respectively. (B) Fusion index was calculated. All the experiments were repeated at least three times. *P < 0.05 and ***P < 0.001.

**Additional file 2: Fig. S2.** Silencing of miR-155 suppresses oxidative stress-induced pyroptosis of trophoblasts in preeclampsia model in vitro by upregulating Nrf2. (A) MiR-155 expression levels were measured using qPCR in HTR-8/SVneo cells after indicated treatments. (B) Nrf2 mRNA levels were measured using qPCR in HTR-8/SVneo cells after indicated treatments. (C) Nrf2 protein levels were measured by western blotting in HTR-8/SVneo cells after indicated treatments. (D) ROS and MDA levels were measured using ROS assay Kit and MDA assay Kit, respectively. (E) Protein levels of HO-1, NQO1 and eNOS were measured by western blotting. (F) Caspase-1, a pyroptosis marker, was measured using flow cytometry. (G) Protein levels of pyroptosis markers (caspase-1, NLRP3, ASC and HMGB1) were measured by western blotting. (H) HMGB1 protein levels were measured using ELISA. All the experiments were repeated at least three times. *P < 0.05, **P < 0.01 and ***P < 0.001.

**Additional file 3: Fig. S3.** Silencing of miR-155 induces function of trophoblasts and M2 macrophage polarization in preeclampsia model in vitro by upregulating Nrf2. (A) M2 macrophage marker (Arg-1, CD68) levels were measured by flow cytometry in macrophages co-cultured with trophoblasts after indicated treatments. (B) Arg-1 expression levels were measured by qPCR in macrophages co-cultured with trophoblasts after indicated treatments. (C) iNOS expression levels were measured by qPCR in macrophages co-cultured with trophoblasts after indicated treatments. (D) Cell proliferation was measured by CCK-8 assay in trophoblasts after indicated treatments. (E) Cell proliferation was measured by colony formation assay in trophoblasts after indicated treatments. (F) Cell migration ability was assessed using wound healing assay in trophoblasts after indicated treatments. (G) Cell invasion ability was measured using Transwell assay in trophoblasts after indicated treatments. All the experiments were repeated at least three times. *P < 0.05, **P < 0.01 and ***P < 0.001.

**Additional file 4: Fig. S4.** MiR-155 silence improves fusion ability of BeWo cells by negatively targeting Nrf2 in preeclampsia model in vitro. (A) After being cultured for 24 h on poly-L-lysine-coated cover glass, BeWo cells with indicated treatments were treated by 30 μM forskolin for 48 h to induce the syncytialization of BeWo cells. Immunofluorescence assay was used to visualize the cell membrane and nucleus with anti-E-cadherin antibody and DAPI, respectively. (B) Fusion index was calculated. All the experiments were repeated at least three times. *P < 0.05, **P < 0.01 and ***P < 0.001.

## Data Availability

The datasets used or analyzed during the current study are available from the corresponding author on reasonable request.

## Abbreviations

Nrf2: Nuclear factor erythroid 2-related factor-2
miR-155: MicroRNA-155
HMGB1: High mobility group box 1
eNOS: Endothelial nitric oxide synthase
LINC00240: Long intergenic non-protein coding RNA 240
ESCC: Esophageal squamous cell carcinoma
ATCC: American Type Culture Collection
UTR: Untranslated region
RIP: RNA binding protein immunoprecipitation
CCK-8: Cell Counting Kit-8
ROS: Oxidative stress
OD: Optical density
LPS: Lipopolysaccharide
CK7: Cytokeratin 7
U6 snRNA: U6 small nuclear RNA
iNOS: Inducible nitric oxide synthase
L-NA: Nu-nitro-l-arginine-methyl ester
