# Supplementary figures and images for: LINC00240/miR-155 axis regulates function of trophoblasts and M2 macrophage polarization via modulating oxidative stress-induced pyroptosis in preeclampsia
Source: Mol Med. 2022 Sep 24;28:119. doi: 10.1186/s10020-022-00531-3 (PMC9509611; doi:10.1186/s10020-022-00531-3)

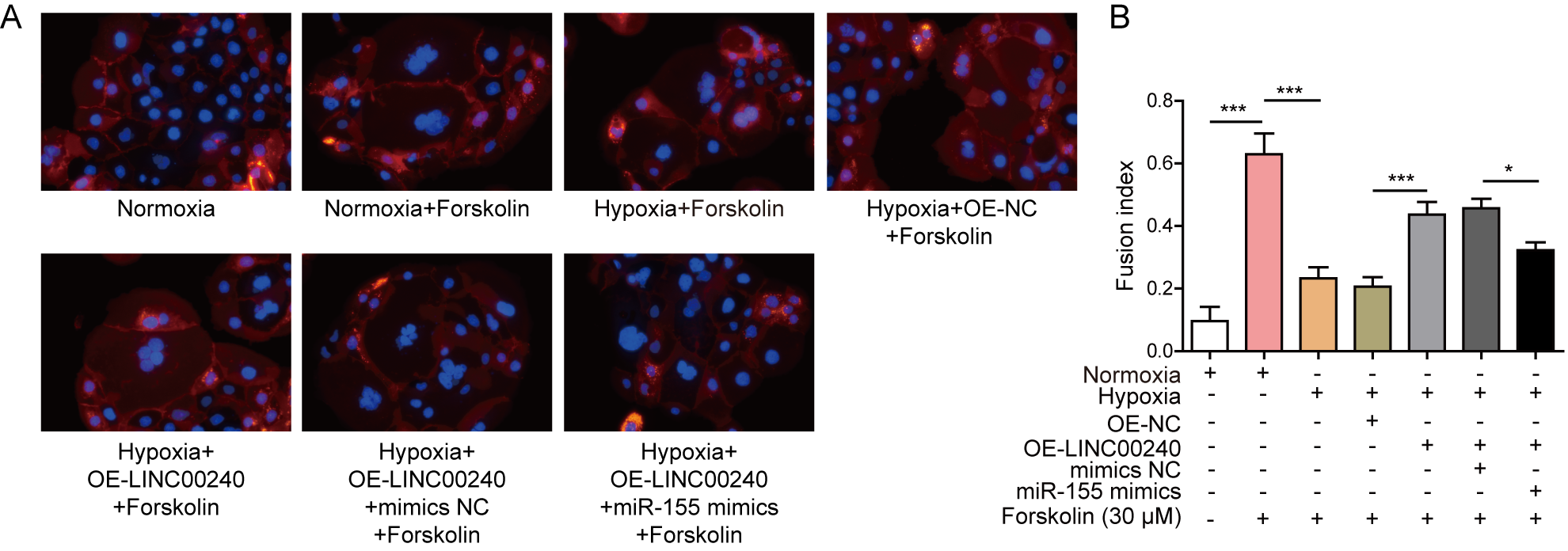

Supplement: Supplementary file 1 — Additional file 1: Fig. S1. LINC00240 improves fusion ability of BeWo cells by negatively targeting miR-155 in preeclampsia model in vitro. After being cultured for 24 h on poly-L-lysine-coated cover glass, BeWo cells with indicated treatments were treated by 30 μM forskolin for 48 h to induce the syncytialization of BeWo cells. Immunofluorescence assay was used to visualize the cell membrane and nucleus with anti-E-cadherin antibody and DAPI, respectively. (B) Fusion index was calculated. All the experiments were repeated at least three times. *P < 0.05 and ***P < 0.001. [file 10020_2022_531_MOESM1_ESM.tif]

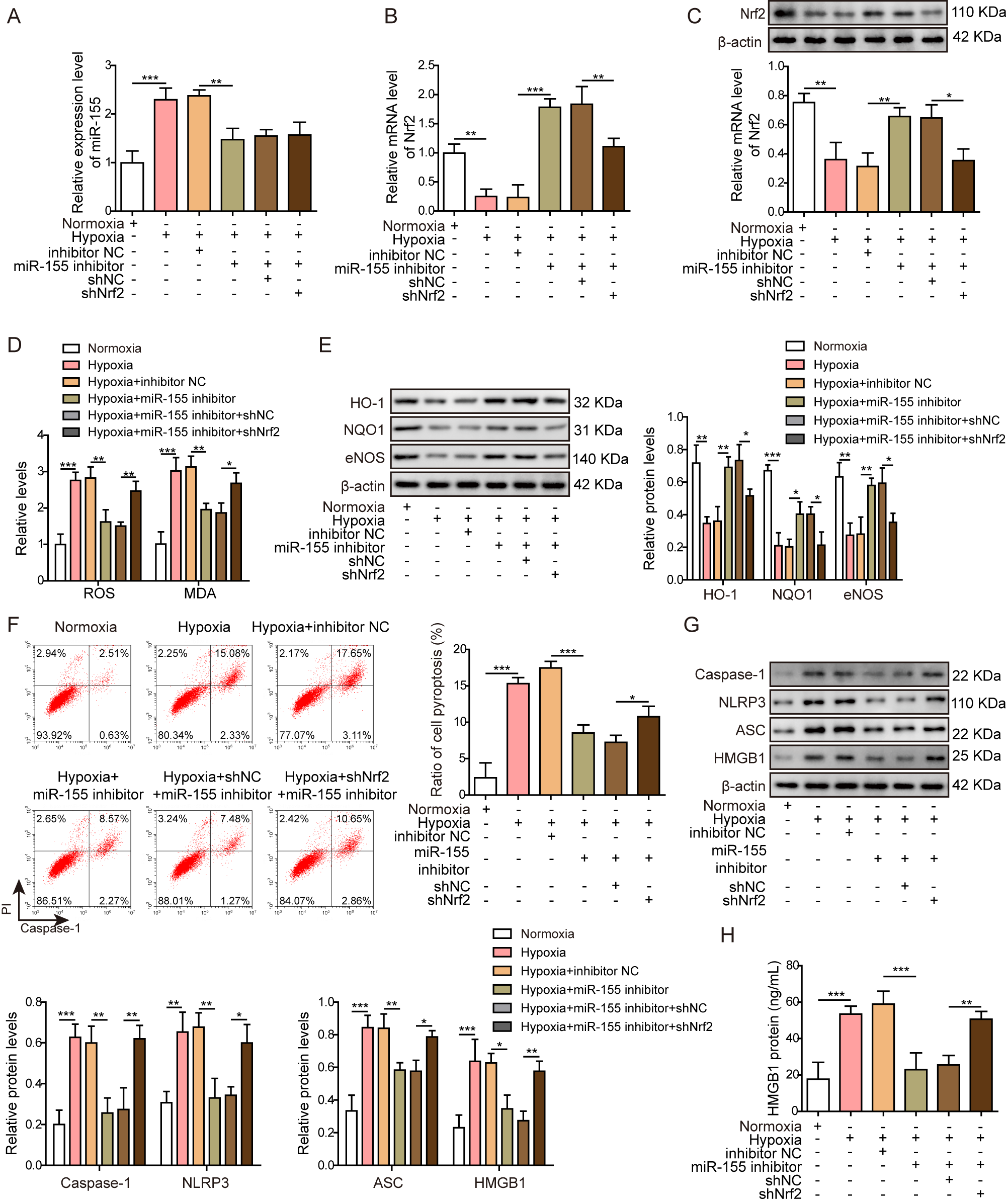

Supplement: Supplementary file 2 — Additional file 2: Fig. S2. Silencing of miR-155 suppresses oxidative stress-induced pyroptosis of trophoblasts in preeclampsia model in vitro by upregulating Nrf2. (A) MiR-155 expression levels were measured using qPCR in HTR-8/SVneo cells after indicated treatments. (B) Nrf2 mRNA levels were measured using qPCR in HTR-8/SVneo cells after indicated treatments. (C) Nrf2 protein levels were measured by western blotting in HTR-8/SVneo cells after indicated treatments. (D) ROS and MDA levels were measured using ROS assay Kit and MDA assay Kit, respectively. (E) Protein levels of HO-1, NQO1 and eNOS were measured by western blotting. (F) Caspase-1, a pyroptosis marker, was measured using flow cytometry. (G) Protein levels of pyroptosis markers (caspase-1, NLRP3, ASC and HMGB1) were measured by western blotting. (H) HMGB1 protein levels were measured using ELISA. All the experiments were repeated at least three times. *P < 0.05, **P < 0.01 and ***P < 0.001. [file 10020_2022_531_MOESM2_ESM.tif]

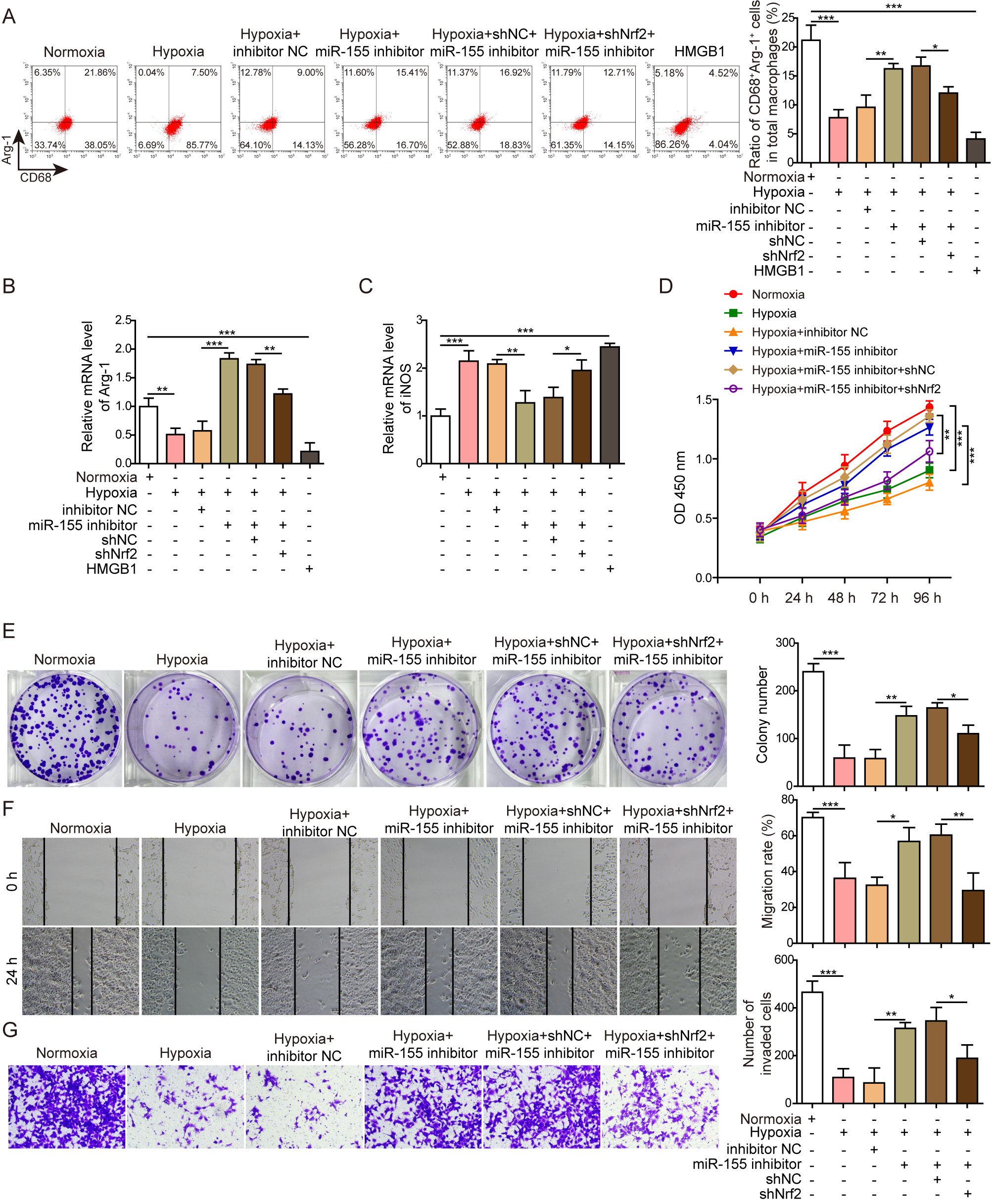

Supplement: Supplementary file 3 — Additional file 3: Fig. S3. Silencing of miR-155 induces function of trophoblasts and M2 macrophage polarization in preeclampsia model in vitro by upregulating Nrf2. (A) M2 macrophage marker (Arg-1, CD68) levels were measured by flow cytometry in macrophages co-cultured with trophoblasts after indicated treatments. (B) Arg-1 expression levels were measured by qPCR in macrophages co-cultured with trophoblasts after indicated treatments. (C) iNOS expression levels were measured by qPCR in macrophages co-cultured with trophoblasts after indicated treatments. (D) Cell proliferation was measured by CCK-8 assay in trophoblasts after indicated treatments. (E) Cell proliferation was measured by colony formation assay in trophoblasts after indicated treatments. (F) Cell migration ability was assessed using wound healing assay in trophoblasts after indicated treatments. (G) Cell invasion ability was measured using Transwell assay in trophoblasts after indicated treatments. All the experiments were repeated at least three times. *P < 0.05, **P < 0.01 and ***P < 0.001. [file 10020_2022_531_MOESM3_ESM.tif]

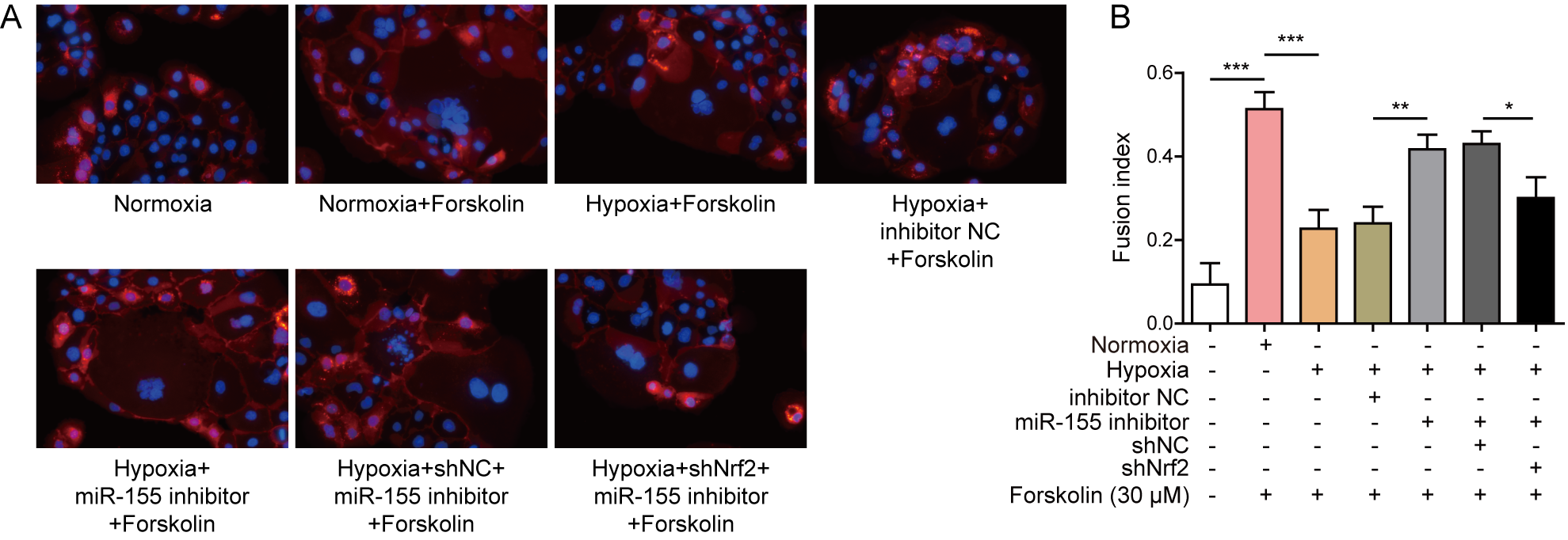

Supplement: Supplementary file 4 — Additional file 4: Fig. S4. MiR-155 silence improves fusion ability of BeWo cells by negatively targeting Nrf2 in preeclampsia model in vitro. (A) After being cultured for 24 h on poly-L-lysine-coated cover glass, BeWo cells with indicated treatments were treated by 30 μM forskolin for 48 h to induce the syncytialization of BeWo cells. Immunofluorescence assay was used to visualize the cell membrane and nucleus with anti-E-cadherin antibody and DAPI, respectively. (B) Fusion index was calculated. All the experiments were repeated at least three times. *P < 0.05, **P < 0.01 and ***P < 0.001. [file 10020_2022_531_MOESM4_ESM.tif]
